# A Descriptive Study of Feelings of Arrested Escape (Entrapment) and Arrested Anger in People Presenting to an Emergency Department Following an Episode of Self-Harm

**DOI:** 10.3389/fpsyt.2016.00155

**Published:** 2016-09-14

**Authors:** Martin Clarke, Kirsten McEwan, Jennifer Ness, Keith Waters, Jaskaran Basran, Paul Gilbert

**Affiliations:** ^1^Research and Development Centre, Kingsway Hospital, Derbyshire Healthcare NHS Foundation Trust, Derby, UK; ^2^Nottinghamshire Healthcare NHS Foundation Trust, University of Nottingham Innovation Park, Nottingham, UK; ^3^Cardiff University School of Medicine, Neuadd Meirionnydd, Heath Park, Cardiff, UK; ^4^Centre for Compassion Research and Training, College of Health and Social Care Research Centre, University of Derby, Derby, UK

**Keywords:** anxiety, depression, self-criticism, shame, entrapment, arrested anger, self-harm, arrested escape

## Abstract

**Background and objectives:**

To explore the role of elevated feelings of anger and desires to escape (fight/flight), which are experienced as inhibited, blocked, and arrested (i.e., arrested anger and arrested flight/escape leading to feelings of entrapment). This descriptive study developed measures of arrested anger and arrested flight and explored these in the context of a recent self-harm event in people presenting to a Hospital’s Emergency Department (ED).

**Methods:**

Fifty-eight individuals presenting to an ED following an act of self-harm were recruited. Participants completed newly developed measures of arrested flight, arrested anger and anger with self in regard to self-harm, and suicide intent and depression.

**Results:**

Ninety-three percent of participants presented after self-poisoning. The majority (95%) reported having experienced high escape motivation that felt blocked (arrested flight) with 69% reporting feeling angry with someone but unable to express it (arrested anger). For many participants (53.7%), strong desires to escape from current situations and/or to express anger did not diminish immediately after the act.

**Limitations:**

As with many studies, a select group of participants agreed to take part and we did not keep records of how many refused. There are no other validated measures of arrested escape and arrested anger and so for this study, our short item-focused measures rely on face validity.

**Conclusion:**

Arrested defenses of fight and flight, and self-criticism are common in those who have self-harmed and may continue after acts of self-harm. Many participants revealed that talking about their experiences of escape motivation and blocked anger (using our measures) was helpful to them.

**Practice points:**

- Feelings of entrapment and arrested anger are common in people who self-harm
- Clinicians could benefit from increased awareness and measures of arrested flight and arrested anger
- Discussing these concepts and experiences appears to be useful to people who have self-harmed
- Further research is needed on how best to help people with such experiences

## Introduction

Suicide accounts for ~8.8 deaths per 100,000 in England (1), with depression being one of its major vulnerability factors (2). The National Suicide Prevention Strategy aims to reduce this rate by monitoring and supporting high risk groups (2). Self-harm is among the strongest risk factors linked to subsequent suicide, although most people who self-harm do not intend to kill themselves (3, 4).

Self-harm can be defined as any intentional act of self-poisoning or self-injury, irrespective of motivation (5). Although most episodes of self-harm do not result in a hospital admission (6), it accounts for over 200,000 attendances to Emergency Departments (EDs) in England every year (7). Many factors, such as chronic physical pain, low self-esteem, relational conflict, bereavement, hopelessness, social isolation, and access to means, are linked to both depression and self-harm (8, 9). In addition, evolution informed concepts of social defeat, blocked escape behavior (entrapment), and blocked arrested aggression have been linked to a range of mental health problems, especially depression (10, 11) and also self-harm and suicide intent (12). A major review of these processes found them to be common underlying mechanisms that link to depression, affect regulation, and urges to self-harm (13). To date, however, there has been no specific measure of entrapment and arrested anger in people who self-harm. This study, therefore, developed a semi-structured interview and explored the degree to which these experiences are related to self-harm, self-hurting behavior, suicide intent, depression, anxiety, and stress in individuals presenting to an ED following an act of self-harm. We hypothesized that these individuals would be highly textured by feelings of escape, preoccupied with fantasies of escape and also feelings of arrested anger and preoccupied with fantasies of anger.

### Evolutionary and Behavioral Approaches

Evolutionary approaches to psychopathology seek to identify possible underlying mechanisms that give rise to particular states of mind, especially those that evolved for dealing with threats (14–17). For example, anxiety is linked to mechanisms of threat detection leading to avoidance and escape behaviors (flight); whereas anger is linked to threat detection leading to confrontational/aggressive behaviors (fight) (14, 18). Complementary to the evolutionary approach are behavioral approaches that seek to identify stimulus and contextual factors that can trigger specific behavioral and defensive repertoires – such as fight–flight (19). One approach that brings these models together in the study of self-harm suggests that mental health problems can arise when innate behavioral defenses are activated, but blocked and, thus, become *arrested defenses, thus* staying aroused but failing to turn off because they cannot be executed (16, 20, 21). One classic paradigm that studies the consequences of blocking animals (e.g., escape behaviors) is learned helplessness. In a typical induction, animals are presented aversive stimuli which they cannot escape. This model has been used to explore depression (22). Under these conditions, individuals stay in high states of threat arousal (linked to the amygdala and hypothalamic–pituitary–adrenal systems) with no resolution by, for example, actually escaping and getting away or fighting (23). Indeed, a large body of evidence suggests that self-harm has a number of functions, including interpersonal functions, and the regulation of negative affect especially anger (24–27). Self-harm may become a way of trying to cope with high levels of anger that are blocked from expression or (interpersonally) effective assertiveness. Self-harm has also been linked to escape behavior (28), such as when high levels of escape motivation are blocked (no escape means or routes) leaving a person feeling trapped, stuck in difficult circumstances and at times defeated.

### Blocked/Arrested Escape/Flight

Entrapment (arrested escape/flight) and defeat are both associated with depression and self-harm (13, 29, 30). Brown and Harris (31) developed life event research in relation to depression and developed the life event interview. Later work found that feeling entrapped in conditions linked to adversity were strongly linked to depression. Gilbert et al. (30) used the life event interview in relation to developing an early measure of arrested anger and arrested flight (entrapment). They found that the majority (88%) of a depressed group wanted to escape from things in their life but were unable to, and felt trapped which they linked to their depression. While for some these feelings came with or as part of depression (though not necessarily causally linked), 39% reported that these experiences *preceded* the onset of depression.

Blocked escape regularly appears as a motivation for suicidal behavior. For example, Baumeister (28) suggested that suicide was an effort to not only escape from difficult things in the world but also painful experiences of self. Williams (32) suggested that there is both a yearning for closeness and attachment (sense of loneliness and feeling cut off from others), but crucially also experiences of defeat and entrapment with no escape. He outlined how these combined experiences give rise to experiences of being in high states of distress but perceiving there was: (1) sense of defeat, (2) no escape, and (3) no rescue. Children who are neglected or abused experience threatening and harmful events, but with no rescue or escape. The people (e.g., parents) who should rescue them are often the perpetrators (33). These early aversive life experiences are known to be linked to elevated risk of depression and self-harm (34). Against this background, self-harming behavior can be seen as a “cry for help,” a “cry of pain,” and “escape from pain” (32). Williams’ *Cry of Pain* model has been developed and tested [e.g., Ref. (35)]. Rasmussen et al. (36) investigated a group of self-harming patients compared with a hospitalized control group and found that the three variables (defeat, no escape, no rescue) were higher in the self-harm group than in the control group. Entrapment and defeat were measured by self-report scales developed by Gilbert and Allan (29) who were investigating the arrested defenses. This paper developed a shorter more specific exploration of entrapment and also arrested anger. Furthermore, the cry of pain and the arrested defenses model have a lot in common. To date, however, the cry of pain model does not address arrested anger.

### Blocked/Arrested Fight/Anger

Anger and fight defenses can also be aroused but blocked, as, for example, in somebody who is fearful of their anger or its consequences for various reasons. This inability to express anger in assertive ways and having a fear of anger has long been associated with depression (37–39). Indeed, both the fear of experiencing and expressing anger has been shown to be associated with clinical depression (40). Elevated anger and fear of expressing anger can persist even after recovery from depression and is associated with anger rumination (41). In an undergraduate sample, Gilbert et al. (42) found that anger rumination, and dwelling on anger memories, was highly associated with both depression and feeling trapped. Gilbert et al. (30) found over 80% of a depressed group experienced arrested anger, with 56% reporting that this began before the depression; a kind of brooding resentment that is not expressed.

In relation to suicidal ideation, Van Elderen et al. (43) found that people who had attempted suicide experienced angry feelings more regularly than a community sample; they also scored higher on a measure of internalized anger, but lower on attempts to control both internalized and externalized anger. Although people may experience leakage of their anger, which may appear as losing of control, they may still carry intense anger that they are unable to express (37–39). In addition, many therapies have drawn attention to experiential avoidance where people try to escape from unpleasant thoughts, feelings, and memories; they are in essence “in-flight” from their own experience [e.g., Ref. (44, 45)] and when they are unable to do so, they can experience feelings of being trapped in these negative experiences (a form of internal arrested flight) “wanting to but unable to” states of mind (29), which has also been referred to as “escape from oneself” (28). In regard to anger, this emotion is also associated with suicidal ideation (24) but individuals can be fearful of experiencing anger and expressing it, hence trying to avoid anger (e.g., arrested fight) (30, 46). This links to the literature on self-criticism that indicates that anger with the self is linked to both depression and self-harm (47), and part of this can be to do with inhibiting anger [for a review, see Ref. (48)] and self-criticism mediates the link between early abusive experiences and self-harm (34).

### Aims

Taken together, the literature strongly indicates that arrested defenses (fight/flight) are associated with suicidal ideation and self-harm. This study builds on earlier measures that will enable more specific assessment of arrested flight and fight in relation to self-harm (30). Hence, one of the aims of the study was to develop a measure for the assessment of these arrested defenses, looking at their intensity and frequency. Second, in order for this measure to become clinically useful, we also explored client experience of discussing these themes. Third, we developed a new self-harming/hurting assessment for this study that measures urges to self-harm, methods, frequency, duration and impulsiveness. Inspection of other scales in this area does not capture these themes.

## Materials and Methods

### Participants

Sixty-four individuals who presented to the [name withheld for anonymity for review] Hospital’s ED following an act of self-harm were recruited to the study by the Mental Health Liaison Team (MHLT) clinicians following a full psychosocial assessment. Four participants had consented to participate, but withdrew, reporting that they felt too unwell to continue or could not concentrate. Two participants were excluded as their episode was later classed as “accidental poisoning” by the assessing clinician. The final sample was, therefore, 58.

Demographics are shown in Table 1. The majority of patients were seen either on the Medical Assessment Unit (24/58; 41.4%) or Ward 101 (23/58; 39.7%). Ward 101 is a non-medical ward where ED attendees can be admitted (often overnight) so they have the opportunity to be assessed by a MHLT clinician.

**Table 1:** **Demographics**.

	Males *N* = 18 *n* (%)	Females *N* = 40 *n* (%)	Total *N* = 58 *n* (%)
**Ethnicity**			
White	18 (100.0)	37 (92.5)	55 (94.8)
Other	–	2 (5.0)	2 (3.4)
Not disclosed	–	1 (2.5)	1 (1.7)
**Age**			
18–29	7 (38.9)	16 (40.0)	23 (39.7)
30–49	6 (33.3)	18 (45.0)	24 (41.4)
50+	4 (22.2)	6 (15.0)	10 (17.2)
Not disclosed	1 (5.6)	–	1 (1.7)
**Marital status**			
Single	7 (38.9)	23 (57.5)	30 (51.7)
In a relationship	8 (44.4)	15 (37.5)	23 (39.7)
Divorced/separated	3 (16.7)	2 (5.0)	5 (8.6)
**Living arrangements**			
On own	5 (27.8)	12 (30.0)	17 (29.3)
With partner	8 (44.4)	11 (27.5)	19 (32.8)
With parent(s)	3 (16.7)	7 (17.5)	10 (17.2)
With children	1 (5.6)	5 (12.5)	6 (10.3)
With other family member	–	2 (5.0)	2 (3.4)
With non-family member	1 (5.6)	2 (5.0)	3 (5.2)
Not disclosed	-	1 (2.5)	1 (1.7)
**Employment status**			
Employed	10 (55.6)	13 (32.5)	23 (39.7)
Unemployed	6 (33.3)	22 (55.0)	28 (48.3)
Carer	1 (5.6)	3 (7.5)	4 (6.9)
Student	1 (5.6)	1 (2.5)	2 (3.4)
Retired	–	1 (2.5)	1 (1.7)
**Location of assessment**			
Ward 101 (non-medical)	8 (44.4)	15 (37.5)	23 (39.7)
Medical Assessment Unit	6 (33.3)	18 (45.0)	24 (41.4)
Other	4 (22.2)	7 (17.5)	11 (19.0)
**Method of self-harm**			
Self-poisoning	15 (83.3)	39 (97.5)	54 (93.1)
Self-injury	2 (11.1)	1 (2.5)	3 (5.2)
Unspecified means	1 (5.6)	–	1 (1.7)

The majority of participants were female (40/58; 69.0%). The mean age for males and females was 36.5 years (SD = 12.9; range 18–53 years) and 36.5 years (SD = 13.6; range 18–71 years), respectively. Age was missing for one male. Of the 23 people who were employed, 4 reported to be on sick leave. Of the 28 who were unemployed, 20 reported to be registered “sick” or “disabled,” with the other 8 currently seeking work.

### Procedure

Following a suspected self-harm presentation, patients considered by the ED or other ward staff to be medically fit enough for psychosocial assessment are referred to the MHLT. Following a full psychosocial assessment, those meeting the study criteria could be asked by the MHLT clinician if they would be willing to meet with a researcher to discuss how different people experience powerful and distressing feelings, in order to better understand and help people who feel very distressed. Whether a patient was asked to participate or not depended upon maintaining patient safety and the research not interfering with ongoing treatment (e.g., if they were to be admitted to a mental health unit).

When the clinician had gained verbal consent, the researcher met with the patient on the ward, mainly in a quiet room to enable for privacy and to ensure confidentiality, to provide them with more details of the study and gain informed consent. The researcher then went through the measures with the participant, which took ~45 min. Participants had the option to complete the measures on their own rather than reading through it with the researcher. Only two participants chose to do so. The researchers were also provided with the Beck Suicide Intent Scale (SIS) scores and ICD-10 codes for the presenting method/s of self-harm, which are routinely recorded by the MHLT clinicians during their psychosocial assessment. Finally, a debrief letter was given to participants thanking them for their participation and reminding them of the telephone support numbers given to them as part of their care plan provided by the MHLT.

Some participants recruited in the early stages of the study expressed that they found participation in the research particularly interesting and beneficial. Consequently, an amendment was made to the ethics application to allow us to capture this information following the completion of the questionnaires. Hence, 27 participants were asked “What was your experience of being involved in this research study?”

### Measures

Participants completed a number of questionnaires related to the study aims. In addition, a range of self-report measures covering, defeat, entrapment, self-concealment, safeness, attachment, depression, anxiety, and stress were completed but are not reported here. In terms of gender, ethnicity, and method of self-harm, the proportions found here were in line with previous self-harm hospital presentation studies (49, 50).

#### Arrested Fight and Flight

This semi-structured interview schedule of questions builds on the scales of Gilbert et al. (30) that measures an individual’s feelings of entrapment and anger, when these began (i.e., before or after depression), their experience of these feelings, the reasons for the desire to escape and anger, and the perceived consequences of escaping or expressing anger. The measure contains 8 escape items and 10 anger items. The escape and anger sections are conditional in that each part is completed following a “yes” response to the respective Question 1. The version used in the present study (Appendix A) was modified to focus on feelings leading up to the episode of self-harm. Four additional questions were added: “How strongly do you want to escape now?”, “How strongly would you rate your feelings of anger now?”, “In general how critical are you of yourself?”, and “In general how angry do you get with yourself?”

#### Self-Hurting Scale and Risk Behavior History

We developed this conditional 11-item semi-structured interview assessment for this study. The measure is conditional in that the respondent omits questions 1b–3 if they respond “0” to question 1a. The schedule measures urges to self-harm, methods, frequency, duration, and impulsiveness (Appendix B). We explored other measures in this area, but felt they were inappropriate for this population and this study.

#### Beck Suicide Intent Scale

The Beck Suicide Intent Scale (51) is a 15-item questionnaire designed to assess the degree of suicidal intent associated with an episode of self-harm. Each item scores 0–2, giving a maximum possible score of 30. Higher scores indicate higher risk. Part I consists of eight items concerning the objective circumstances of the self-harm act, for example, precautions against discovery or intervention. Part II consists of seven self-report items based on the patients’ own recollection of their feelings and thoughts at the time of the act, for example, the alleged purpose of attempt. The Cronbach’s alpha in our study for the total, circumstances, and self-report subscales were 0.84, 0.66, and 0.99, respectively.

#### Depression, Anxiety, and Stress Scale (DASS-21)

This 21-item shortened version of the Depression, Anxiety, and Stress Scale (DASS-42) comprises three subscales measuring *Depression* (e.g., “I felt I wasn’t worth much”), *Anxiety* (e.g., “I felt close to panic”), and *Stress* (e.g., “I found myself agitated”) (52). Participants are asked to rate how much each statement applied to them over the past week, on a four-point Likert scale from 0 (“Does not apply to me at all”) to 3 (“Applied to me very much, or most of the time”). We correlated key variables with the DASS. Otherwise, the DASS is reported elsewhere. The Cronbach’s alpha in our study for the Depression, Anxiety, and Stress subscales were 0.96, 0.84, and 0.99, respectively.

### Ethics

The study was granted ethical approval from the local NHS research ethics committee, as well as the Research and Development departments of both the mental health and acute NHS Trusts.

### Statistical Analyses

Analyses and calculations were performed with SPSS (PASW) version 18. Reliability analyses were conducted reporting Cronbach’s alpha where appropriate. For scales that comprised items with different response formats and where items could not be summed, face validity was assessed. Descriptive analyses were conducted and the mean, SD, and range reported for continuous variables. Gender differences in scores were assessed using *t*-tests or Fisher’s exact test. For scales comprising individual items, frequency analysis was conducted and percentages were reported. To assess the relationship between variables and to test the hypotheses, non-parametric Spearman’s Rho correlations were performed.

We hypothesized that desires to escape and feelings of anger would be high in this population; hence, we conducted descriptive analyses and frequency analyses to test this hypothesis. We also hypothesized that these desires to escape and feelings or anger would be associated with depression and self-harming behaviours; to test this, a correlation analysis was conducted. Finally, we hypothesized that in line with previous literature concerning gender differences and suicide, that males would have greater suicide intent than females (53), there would be no difference in entrapment (29), and anger would be higher in males (54).

## Results

### Method of Self-Harm

The majority of participants presented after self-poisoning (54/58; 93.1%) with only a few presenting after self-injury (3/58; 5.2%). The method for one individual was recorded as self-harm by unspecified means (1.7%).

### Suicidal Intent

The mean total SIS score was 9.4 (*SD* = 5.4; range 1–28). The mean circumstances score was 3.5 (SD = 2.6; range 0–14) and the mean self-report score was 5.9 (*SD* = 3.5; range 0–14). There was no difference between the mean total SIS scores for males and females, 10.6 (SD = 5.5) and 8.9 (SD = 5.3), respectively [*t*(56) = 1.07, *p* > 0.05].

### Escape

The majority of participants (55/58; 94.8%) reported that prior to their self-harm episode, they felt they wanted to escape and get away from things. The three participants who reported not wanting to escape did not complete the remaining escape questions. Over two-thirds (70.9%; 39/55) reported feeling this way for at least 6 months (10 of whom had felt like escaping for over 5 years); 16.4% (9/55) for between 1 and 6 months; 10.9% (6/55) for less than 1 month; and 1.8% (1/55) did not answer.

Using a 6-point Likert scale from 0 (not at all) to 5 (very strongly), 80% of participants reported their desire to escape leading up to their episode of self-harm as at least a “4”: 45.5% (25/55) and 34.5% (19/55) rated the desire as “5” or “4”, respectively. A further 18.2% (10/55) rated the desire as “3” and 1.8% (1/55) as “2.” Females rated their desire to escape (M = 4.40; SD = 0.76) significantly stronger than males (M = 3.81; SD = 0.83) *t*(53) = −2.52, *p* < 0.05. When the length of time participants had felt like escaping was categorized into less than 6 months or 6 months and over, significantly more females than males had felt like escaping for longer (Fisher’s exact test, *p* < 0.05).

When asked how strongly they wanted to escape now, approximately half of all participants reported their desire to escape as at least a “4”: 40.7% (22/54) and 13.0% (7/54) rated the desire as “5” or “4”, respectively. Ratings of “3,” “2,” “1,” or “0” were given by 22.2% (12/54), 5.6% (3/54), 9.3% (5/54), and 9.3% (5/54), respectively, with one participant not responding. Approximately only half (48.1%, 26/54) reported a decrease in their desire to escape now. Approximately one-third (37.0%, 20/54) reported no change. For 14.8% (8/54), the desire to escape had increased after their episode of self-harm.

When presented with a 12-item tick list, participants identified a range of people or situations that they wanted to escape from. The most common was their own thoughts and feelings (44/54; 83.0%). Other sources of escape desires included: isolation (31/54; 57.4%); money problems (32/54; 59.3%); an illness (25/54; 46.3%); parents (19/54; 35.2%); family (18/54; 33.3%); job (18/54; 34.0%); partner (10/54; 18.5%); friends (7/54; 13.0%); neighbors (7/54; 13.0%); children (3/54; 5.7%); and other (10/55; 18.2%). One participant only ticked “other” and specified “everything” and as this could be a generalization was not included in the denominator for the individual sources.

Furthermore, the majority of participants reported that they frequently felt like and fantasized about escaping but few made specific plans to (Table 2). No significant differences were found in participants’ desire to escape based on SIS score.

**Table 2:** **Frequencies for Escape and Anger inhibition**.

Escape and anger inhibition		*N* (%)
**Escape**		
Felt like escaping and getting away prior to episode of self-harm?	Yes	55 (94.8)
	No	3 (5.2)
Change in desire to escape now compared to at time of self-harm	Higher now	8 (14.8)
	No change	20 (37.0)
	Lower now	26 (48.1)
How often did you feel like escaping?	Not at all	1 (1.8)
	A little	6 (10.9)
	Quite a lot	26 (47.3)
	Most of the time	22 (40.0)
How often did you fantasize about escaping?	Not at all	4 (7.4)
	A little	11 (20.4)
	Quite a lot	20 (37.0)
	Most of the time	19 (35.2)
How often did you make specific plans to escape?	Not at all	16 (29.1)
	A little	15 (27.3)
	Quite a lot	19 (34.5)
	Most of the time	5 (9.1)
**Anger**		
Felt angry with someone but unable to tell them, prior to self-harm?	Yes	40 (69.0)
	No	18 (31.0)
Change in feelings of anger now compared to at time of self-harm	Higher now	3 (7.5)
	No change	13 (32.5)
	Lower now	24 (60.0)
How often did you feel angry?	Not at all	–
	A little	7 (17.5)
	Quite a lot	19 (47.5)
	Most of the time	14 (35.0)
How often did you fantasize about telling others how angry or irritated you were with them?	Not at all	2 (5.0)
	A little	8 (20.0)
	Quite a lot	18 (45.0)
	Most of the time	12 (30.0)
How often did you make specific plans to tell others how angry or irritated you were with them?	Not at all	9 (22.5)
	A little	9 (22.5)
	Quite a lot	14 (35.0)
	Most of the time	8 (20.0)
In general how critical are you of yourself?	Not at all	2 (5.0)
	A little	6 (15.0)
	Quite a lot	12 (30.0)
	Most of the time	20 (50.0)
In general how angry do you get with yourself?	Not at all	2 (5.0)
	A little	3 (7.5)
	Quite a lot	13 (32.5)
	Most of the time	22 (55.0)

### Anger

Over two-thirds of participants (40/58; 69.0%) reported that prior to their self-harm episode, there were occasions when they felt angry with someone but were unable to tell them. The 18 participants who reported not feeling angry did not complete the remaining anger questions. Almost two-thirds (25/39; 64.1%) reported feeling this way for at least 6 months; 27.5% (11/39) for between 1 and 6 months, 7.7% (3/39) for less than 1 month and one individual did not answer.

Participants were asked whether they had a strong wish to tell others how angry or irritated they were with them in the period leading up to their episode of self-harm. Using a 6-point Likert scale from 0 (not at all) to 5 (very strongly), the majority rated the desire as at least a “4”: 52.5% (21/40) and 35.0% (14/40) rating their desire as “5” or “4”, respectively. A further 9.5% (3/40) rated the desire as “3,” 2.5% (1/40) as “2,” and 2.5% (1/40) as “0.” No difference was found between males (M = 4.18; SD = 0.75) and females (M = 4.34; SD = 1.11) in their rating of how strongly they wanted to tell others about their anger [*t*(38) = −0.45, *p* > 0.05].

When asked to rate how strongly their feelings of anger were now, the majority of participant’s responses indicated that their feelings had become less strong than those felt prior to the self-harm act: 15.0% (6/40) rated their current feelings of anger as “0,” 7.5% (3/40) as “1,” 22.5% (9/40) as “2,” 12.5% (5/40) as “3,” 17.5% (7/40) as “4,” and 25.0% (10/40) as “5.” Indeed, the responses of 60.0% (24/40) of participants indicated a decrease in feelings of anger, with 32.5% (13/40) of participants indicating that they were still as angry as before their self-harm episode and 7.5% (3/40) of participant’s responses indicated that their feelings of anger had increased.

When presented with a 12-item tick list, participants identified a range of people or situations that they were angry with. The most common were their own thoughts and feelings (26/40; 65.0%) and money problems (21/40; 52.5%). Other sources of anger included: isolation (17/40; 42.5%); an illness (17/40; 42.5%); parents (18/40; 45.0%); family (16/40; 40.0%); job (14/40; 35.0%); partner (16/40; 40.0%); friends (15/40; 37.5%); neighbors (6/40; 15.0%); children (6/40; 15.0%); and other (10/40; 25.0%).

When participants were asked how often they felt like, fantasized about, and planned to tell others how angry they were with them, feelings of anger and fantasies of telling others were frequent. However, few made specific plans to tell others how angry they were with them. The majority of participants also reported being self-critical and/or angry with themselves “quite a lot” or “most of the time” (Table 2). No significant differences were found in feelings of anger based upon SIS score.

Overall, 39 (67.2%) participants experienced feelings of both “anger with someone” and “wanting to escape” leading up to the episode of self-harm. Of these, 28 participants (48.3% of the sample) reported the strength of these feelings was at least a “4” on both anger and escape (scales from “0” not at all to “5” very strongly).

### Self-Hurting and Risk Behavior

Participants were asked how often they had urges to hurt themselves using a 6-point Likert scale from 0 (never) to 5 (a lot of the time). Approximately half (48.3%, 28/58) rated the frequency of urges as either a “5” or a “4,” with 10.3% (6/58) reporting to never feel urges (see Table 3). For the majority of those who reported to have had urges to hurt themselves, their urges were long-standing. Indeed 78.8% (41/52) had felt urges for at least 6 months, and 30.8% (16/52) had felt this way for over 5 years. When asked how often they actually hurt themselves, 17.3% (9/52) of participants reported that they never hurt themselves. However, when asked how long they had done this, three of these nine participants responded in a way which indicated that they had previously hurt themselves.

**Table 3:** **Frequencies for self-hurting and risk behavior**.

Self-hurting and risk behavior questions		*n*/*N* (%)
Have you felt urges to hurt yourself?	0 (never)	6 (10.3)
	1	5 (8.6)
	2	7 (12.1)
	3	12 (20.7)
	4	8 (13.8)
	5 (a lot of the time)	20 (34.5)
How long have you felt like this?	Less than 1 month	3 (5.8)
	1–6 months	8 (15.4)
	Over 6 months	41 (78.8)
How often do you hurt yourself?	Never	9 (17.3)
	Rarely	12 (23.1)
	Sometimes	19 (36.5)
	Often	9 (17.3)
	Very often	3 (5.8)
How long have you done this?	Never^a^	6 (11.5)
	Less than 1 month	3 (5.8)
	1–6 months	6 (11.5)
	Over 6 months	37 (71.2)
How do you hurt yourself? (open question)	Cutting	29 (50.0)
	Overdose	34 (58.6)
	Substance abuse	2 (3.4)
	Physical abuse	12 (20.7)
	Mental abuse	1 (1.7)
	Toxic substance (bleach)	1 (1.7)
	Hanging	3 (5.2)
	Dangerous risk	1 (1.7)
Have you acted in ways which put yourself at risk (e.g., drinking too much or driving too fast or dangerously)?	0 (never)	11 (19.0)
	1	6 (10.3)
	2	5 (8.6)
	3	9 (15.5)
	4	9 (15.5)
	5 (a lot of the time)	18 (31.0)
How long have you done this?	Less than 1 month	1 (2.1)
	1–6 months	4 (8.5)
	Over 6 months	39 (83.0)
	Missing	3 (6.4)
Have you attempted suicide before?	Yes	36 (62.1)
	No	22 (37.9)

*^a^Three of the nine gave times rather than “never”*.

Some participants reported to have previously used several methods of self-harm, although self-poisoning and cutting were the most commonly reported. Self-poisoning and cutting was also the most common combination with 34.6% (18/52) reporting to have used both methods of self-harm.

The majority of participants (81.0%, 47/58) reported that they had acted in ways which put themselves at risk, with almost one-third (31.0%, 18/58) rating the frequency as “a lot of the time.” The majority of those that did put themselves at risk had done so for at least 6 months (83.0%, 39/47). Participants were asked whether they had “attempted suicide before.” It should be acknowledged that this could be interpreted differently by different people. However, the responses indicate that previous “suicide attempts” were common with 62.1% (36/58) reporting to have “attempted suicide” before. Of these, 70.2% (26/37) reported they had made more than 1 attempt; 1 person stated they had made over 30 attempts. No significant differences were found between the SIS scores in relation to self-hurting/risk behavior.

### Arrested Fight/Flight and Psychopathology, Suicide Intent, and Self-Hurting

Table 4 gives the relationship of arrested defenses with psychopathology, suicide intent, and self-hurting. Feeling angry with the self and self-criticism were both significantly linked to depression. Feelings of escape and plans of escape were significantly linked with anxiety and stress. Suicidal intent was not associated with arrested defenses, however, urges to hurt oneself was significantly associated with arrested defenses.

**Table 4:** **Spearman’s rho correlations between depression, anxiety, stress, suicide intent self-harm with arrested fight, flight, and self-critical measures**.

	Depression	Anxiety	Stress	Suicide intent	Urges to hurt self
How often did you feel like escaping	0.23	0.30^a^	−0.05	0.04	0.43^b^
How often did you make specific plans to escape	0.26	0.39^b^	0.37^b^	−0.16	0.40^b^
How often did you feel angry	0.18	0.21	0.07	0.17	0.44^b^
How often did you make plans to tell others about anger	−0.02	0.04	0.02	−0.11	0.23
How critical are you of yourself	0.45^b^	0.39^a^	0.19	0.05	0.39^a^
How angry are you with yourself	0.51^b^	0.41^b^	0.28	−0.04	0.41^b^

*^a^Correlation is significant at the 0.05 level (two-tailed)*.

*^b^Correlation is significant at the 0.01 level (two-tailed)*.

*Depression, depression from the DASS; anxiety, anxiety from the DASS; stress, stress from the DASS; suicide intent, Beck Suicide Intent Scale; urges to hurt self, self-hurting scale and risk behavior history; and other items, arrested fight and flight scale*.

### Participant Experience

In order to evaluate how clients experience discussing these themes, which are not typical of clinical interview, we asked 27 participants 1 open-ended question about their experience of being involved in the research study. The majority reported that they found it to be a positive experience. Participants appeared to feel empowered by the thought that they may be able to help others going through similar experiences:

“Overall a positive experience… I wanted to help other people understand self- harm” [32].

Feedback also indicated that the research had provided some with a form of validation:

“First time somebody’s sat and asked me how I felt today…helped me to go back over my feelings…would like to thank the researchers for taking the time to talk to me” [37].

“Good questions…related to me…no-one’s ever asked me those questions before” [56].

Participants also reported to have felt that they had directly benefited just through the research process itself:

“Overall positive…the questions made me realise things about myself…made me realize my family do care about me” [31].

“Allows you to vent views” [52].

The research process may have been therapeutic, as it enabled individuals to step back and view their situation more objectively:

“Insight into self” [48].

“Made me think about things more, when you read all the questions and I looked at my scores. Made me realise I am a very unhappy person and I need to do something about it” [65].

Only one participant expressed negative feelings toward the research:

“Made me feel anxious, shaking, clammy hands, relive emotions…brought it all back” [60].

A number of individuals made specific comments in relation to the questions and interview process itself:

“Good, needed to open up, easy to give numbers…feel better after yesterday…given another chance, feel able to be more open and talk about experiences” [27].

“Straight forward…well laid out…sensitive wording…not too deep…quite confidential” [38].

“Questions really did apply to me…any research I am all for it” [62].

“Happy to help…it was helpful…questions not too probing” [23].

Two participants made negative comments in relation to the layout and structure of the questions:

“Alright…confusing scales” [53].

“The questions felt airy fairy” [35].

## Discussion

The aim of this study was to develop a measure for the assessment of arrested defenses, explore client experience of discussing these themes, and develop a new self-harming/hurting assessment as inspection of other scales in this area suggested that they do not capture these themes. The focus and the participant inclusion focused on self-harm rather than suicide intent. Aroused and arrested defenses, in the form of chronic desires to escape and/or to express anger, appear common in those presenting to hospital with self-harm. Furthermore, for nearly half of all participants these strong desires *did not* appear to diminish immediately after the act. Not only does this support the association between arrested defenses with self-harm, it highlights the value of addressing them even after crisis point. It is possible that if feelings of anger and escape are not resolved, individuals can remain in a high state of arousal, ruminating, and fantasizing on these themes, and remain at risk of repeat self-harm. Our data suggest that it is not only feeling trapped in, and angry with, outside situations or people, but also with one’s own thoughts and sense of self; the notion of escape from oneself, or at least one’s thoughts and feelings is supported here (28, 29). However, to fully explore this, this would require comparison with a non-self-harming control group.

Our data show that in this population, 95% of people felt like escaping before their episode of self-harm, and the majority felt like this “quite a lot or most of the time.” In addition, 72.2% fantasized about escaping “quite a lot or most of the time.” Ruminating and fantasizing about flight/escapes will maintain high arousal in the flight (e.g., amygdala and hypothalamic-pituitary adrenal) systems (30). In regard to fight, our data are similar with 69% acknowledging feeling angry with someone that they could not express it to. 82.5% of people felt this “quite a lot or most of the time.” Seventy-five percent fantasized about expressing anger “quite a lot or most of the time.” In regard to *self-directed anger*, 87.5% acknowledged feeling this way toward themselves quite a lot or most of the time. So again the anger system appears to be constantly stimulated through processes of ruminating about anger with others and/or feeling angry with oneself. Most participants reported that there were “numerous things” that they wanted to escape from or felt angry about. In addition, most participants reported wanting to escape from their “own thoughts and feelings.” This is in tune with Baumeister’s (28) concept of escape from the self.

We also draw attention to the fact that 14.8% had a greater desire to escape *after the event* and 37.0% felt no change in their desire to escape. Hence, 51.8% still felt flight motivated. This clearly has implications not only for self-harm but also for depression. In regard to anger, 7.5% of people who felt angry with someone, felt even *angrier* after the event, and 32.5% felt no change. These findings obviously have implications for depression and case and risk management.

We also developed the *Self-Hurting Scale and Risk Behavior History* semi-structured interview assessment that can be used not only for actual self-harm incidents, but for other mental health problems associated with self-harm. The feedback we had from participants was that they understood the questions and were not stressed by responding to them. This assessment allows clinicians to look in detail at various aspects of self-harm, including desire and motivation. In this population, 83% of the sample reported they had self-harmed before. Of these 23.1% reported to have self-harmed “rarely,” 36.5% as “sometimes,” 17.3% “often” and 5.8% “very often.” We are not able to say how much these previous self-harm attempts were related to ongoing unresolved fight and flight issues or depression but this is important to explore, because previous self-harm is one of the strongest predictors for future self-harm and suicide (5, 7, 49). Hence, assessing arrested defenses may be as important a predictor as suicidal intent for subsequent risk of self-harm.

We also explored the relationship between arrested fight and flight, and suicide intent and self-hurting/harming in this population (Table 4). Interestingly, these arrested defenses are linked to self-harm but not suicide intent. It is possible, however, that suicide intent may have reduced subsequent to the episode. Arrested defenses were also linked to anxiety and stress, and self-criticism and anger were associated with depression.

### Clinical Implications

This is the first study to develop specific measures for arrested flight and fight, arrested anger and escape, and dynamics of self-hurting/harming for a self-harming hospital assessed population. The findings have a number of clinical implications. First, it is possible to assess people’s degree of these evolved behavioral defenses namely arrested fight and flight with measures such as these. The degree to which the fight/flight systems (linked to the amygdala and hypothalamic–pituitary–adrenal systems) are activated offers different insights to that of hopelessness (23). There may be many situations that we feel hopeless about but are not in states of arrested fight or flight – for example, develop acceptance or tolerance.

Although potentially painful, importantly most participants felt that systematically going through the experiences of arrested flight/entrapment and fight/anger helped them to make sense of their episode and articulate their feelings. They were given a language that enabled them to think about what they were feeling and to recognize states of mind that might have been difficult to articulate without having those questions or framework. Interestingly, the exploration of their feelings of anger and escape often led to a sense of empowerment, validation, and a deeper insight into their current situation. Simply knowing that arrested escape and anger can be common for people who self-harm offers a sense of common humanity and help to reduce the shame of “it’s only me.” The importance of sensitively but also informatively (e.g., with insight into self-harm) talking through a person’s situation using psychosocial assessments has been repeatedly demonstrated to reduce the risk of repeat self-harm and suicide (3, 55) and so adding these dimensions to discussion could help further. One reviewer of the paper noted that there may be a significant difference between participants reading the questionnaires and answering the questions themselves rather than the researcher reading it to them. The latter may be experienced in a more supportive way. To the best of our knowledge, there are no data on this interesting area.

### Limitations

Recruiting patients from the ED following a presentation of self-harm has been used in numerous previous studies (36, 56). The present study was with a subgroup of patients whom presented to ED following self-harm. However, it is unknown how these findings might generalize to those who are not clinically assessed. To be included in the study, patients had to be over 18 years of age; assessed by an MHLT clinician between 8:00 a.m. and 4:00 p.m.; willing to remain in hospital for a further 30–60 min to see the researcher; not be deemed high risk, i.e., actively suicidal or detained under the Mental Health Act, or in the process of being admitted to the mental health unit. Therefore, a high risk, vulnerable group of people that did not meet the inclusion criteria were not included in this study. However, in terms of gender, ethnicity, and method of self-harm, the proportions found here were approximately in line with those of previous self-harm hospital presentation studies (49, 50).

Our sample size is limited and this is likely due to patients feeling too ill to participate (93% had self-poisoned). Furthermore, some of the MHLT clinicians were hesitant to recruit patients due to a protective instinct to not cause the patient any more distress at such a vulnerable time. When the researchers began to share their positive feedback (that the majority of participants indicated that they found participating to be a positive experience), the referral rate seemed to increase. In addition, we were using clinicians unfamiliar with research and future studies would benefit from more research aware clinicians. We tried to address this limitation by keeping recruitment open for 2 years. It is a further limitation that the clinicians did not record how many patients declined to participate, we, therefore, cannot know how willing this patient group were to engage in research at such a distressing time and cannot report the demographics of those who declined to participate.

## Conclusion

This research suggests that it may be helpful to explore the state of evolved behavioral defenses, with people who self-harm. Problems precipitating the self-harm are unlikely to be resolved if the underlying threat (fight/flight) system remains activated. Obvious examples are of people who feel trapped in relationships they cannot get out of, or in jobs or financial situations they cannot get away from. In addition, some may be replays of childhood experiences of feeling trapped in adverse contexts. The exploration of such underlying mechanisms may also be important for identifying appropriate interventions.

## Author Contributions

MC – data analysis and study coordination; KM – data analysis; JN – data gathering; KW – advisor and study coordination; JB – data analysis; and PG – study designer and coordinator.

## Conflict of Interest Statement

The authors declare that the research was conducted in the absence of any commercial or financial relationships that could be construed as a potential conflict of interest.

## Appendix

### A. Arrested Fight and Flight Scale

#### Escape

When some people become distressed they may want to escape, or get away from things in their life. Below are a series of questions to explore your feelings about this.

1. Leading up to your episode of self-harm, did you feel there were occasions when you felt like escaping and just getting away from things?
   Yes □ No □
  (If no please go to anger inhibition questions on the next page)
2. Roughly how long had you felt like this? …….….years ………months
3. How strongly did you want to escape? (please circle)
  Not at all 0 1 2 3 4 5 Very strongly
4. How strongly do you want to escape now?
  Not at all 0 1 2 3 4 5 Very strongly
5. Who or what did you want to escape from? (Please tick all that apply)

**Table:**

	Yes	No
Friends		
Partner		
Parents		
Children		
Family		
Neighbors		
Isolation		
Job		
Money Problems		
An Illness		
My thoughts and feelings		
Others (please state)		

**Table:**

		Not at all	A little	Quite a lot	Most of the time
6.	How often did you feel like escaping?				
7.	How often did you fantasize about escaping?				
8.	How often did you make specific plans to escape?				

#### Anger

When some people become distressed they may feel angry, frustrated or irritable. Below are a series of questions to explore your feelings about this.

1. Leading up to your episode of self-harm, did you feel there were occasions when you felt angry with someone but were unable to tell them?
   Yes □ No □
  (If no please go to the next questionnaire)
2. Roughly how long had you felt like this? …….….years ………months
3. How strongly did you wish you could tell others how angry or irritated you are with them, but felt unable to? (please circle)
  Not at all 0 1 2 3 4 5 Very strongly
4. How strongly would you rate your feelings of anger now?
  Not at all 0 1 2 3 4 5 Very strongly
5. Who or what were you mainly angry with? (Please tick all that apply)

**Table:**

	Yes	No
Friends		
Partner		
Parents		
Children		
Family		
Neighbors		
Isolation		
Job		
Money Problems		
An Illness		
My thoughts and feelings		
Others (please state)		

**Table:**

		Not at all	A little	Quite a lot	Most of the time
6.	How often did you feel angry?				
7.	How often did you fantasize about telling others how angry you were with them?				
8.	How often did you make specific plans to tell others how angry or irritated you were with them?				
9.	In general how critical are you				
10.	In general how angry do you get with yourself?				

### B. Self-Hurting and Risk Behavior History

When people are distressed they can sometimes want to hurt themselves in various ways. These may include hitting, pinching, cutting, burning, and hair pulling. Below are a series of questions about feelings and behaviors related to self-hurting. Please circle the number that applies to you.

1a. Have you felt urges to hurt yourself? (If answer is no please go to question 4)
  No, never 0 1 2 3 4 5 A lot of the time
1b. How long have you felt like this? …….….years ………months
2a. How often do you hurt yourself? (Please circle)
  **Table:**
  Never	Rarely	Sometimes	Often	Very Often
2b. How long have you done this? …….….years ………months
3. How do you hurt yourself?
4a. Have you acted in ways that put yourself at risk? (For instance, drinking too much, driving too fast or dangerously)
  No, never 0 1 2 3 4 5 A lot of the time
4b. How long have you done this? …….….years ………months
5a. Have you attempted suicide before? Yes □ No □
5b. If YES, how many times before?
5c. When was the first time? …….….years ………months
5d. When was the last time (if more than one)? …….….years ………months
